# Mild behavioral impairment in early Alzheimer’s disease and its association with *APOE* and *BDNF* risk genetic polymorphisms

**DOI:** 10.1186/s13195-024-01386-y

**Published:** 2024-01-26

**Authors:** Veronika Matuskova, Katerina Veverova, Dylan J. Jester, Vaclav Matoska, Zahinoor Ismail, Katerina Sheardova, Hana Horakova, Jiri Cerman, Jan Laczó, Ross Andel, Jakub Hort, Martin Vyhnalek

**Affiliations:** 1 Department of Neurology, Memory Clinic, Charles University, Second Faculty of Medicine and Motol University Hospital, V Uvalu 84, 150 06 Prague, Czech Republic; 2 International Clinical Research Center, St. Anne’s University Hospital, Brno, Czech Republic; 3 Women’s Operational Military Exposure Network (WOMEN), VA Palo Alto Health Care System, Palo Alto, CA USA; 4 Department of Clinical Biochemistry, Hematology and Immunology, Homolka Hospital, Prague, Czech Republic; 5 Departments of Psychiatry and Clinical Neurosciences, Cumming School of Medicine, Hotchkiss Brain Institute, University of Calgary, Calgary, AB Canada; 6 Clinical and Biomedical Sciences, Faculty of Health and Life Sciences, University of Exeter, Exeter, United Kingdom; 7 Department of Clinical Psychology, Motol University Hospital, Prague, Czech Republic; 8 Center for Innovation in Healthy and Resilient Aging, Edson College of Nursing and Health Innovation, Arizona State University, Phoenix, AZ USA

**Keywords:** Alzheimer’s disease, Neuropsychiatric symptoms, Mild behavioral impairment, Mild cognitive impairment

## Abstract

**Background:**

Mild behavioral impairment (MBI) has been commonly reported in early Alzheimer’s disease (AD) but rarely using biomarker-defined samples. It is also unclear whether genetic polymorphisms influence MBI in such individuals. We thus aimed to examine the association between the cognitive status of participants (amnestic mild cognitive impairment (aMCI-AD) vs cognitively normal (CN) older adults) and MBI severity. Within aMCI-AD, we further examined the association between APOE and BDNF risk genetic polymorphisms and MBI severity.

**Methods:**

We included 62 aMCI-AD participants and 50 CN older adults from the Czech Brain Aging Study. The participants underwent neurological, comprehensive neuropsychological examination, APOE and BDNF genotyping, and magnetic resonance imaging. MBI was diagnosed with the Mild Behavioral Impairment Checklist (MBI-C), and the diagnosis was based on the MBI-C total score ≥ 7. Additionally, self-report instruments for anxiety (the Beck Anxiety Inventory) and depressive symptoms (the Geriatric Depression Scale-15) were administered. The participants were stratified based on the presence of at least one risk allele in genes for APOE (i.e., e4 carriers and non-carriers) and BDNF (i.e., Met carriers and non-carriers). We used linear regressions to examine the associations.

**Results:**

MBI was present in 48.4% of the aMCI-AD individuals. Compared to the CN, aMCI-AD was associated with more affective, apathy, and impulse dyscontrol but not social inappropriateness or psychotic symptoms. Furthermore, aMCI-AD was related to more depressive but not anxiety symptoms on self-report measures. Within the aMCI-AD, there were no associations between *APOE* e4 and *BDNF* Met and MBI-C severity. However, a positive association between Met carriership and self-reported anxiety appeared.

**Conclusions:**

MBI is frequent in aMCI-AD and related to more severe affective, apathy, and impulse dyscontrol symptoms. *APOE* and *BDNF* polymorphisms were not associated with MBI severity separately; however, their combined effect warrants further investigation.

**Supplementary Information:**

The online version contains supplementary material available at 10.1186/s13195-024-01386-y.

## Background

Alzheimer’s disease (AD) is characterized by progressive cognitive decline and loss of functional capacity at the stage of dementia [1]. In its prodromal stage, mild cognitive impairment (MCI), there is already objective evidence of cognitive impairment, but daily functioning is generally preserved [2]. To refine the MCI due to AD clinical diagnosis, AD biomarkers are used. Evidence of β-amyloid pathology is essential to the diagnosis of Alzheimer’s pathological change and can be measured in the cerebrospinal fluid (CSF) or by β-amyloid positron emission tomography (PET) imaging. This move to a biological definition is also reflected in the AT(N) diagnostic criteria [3].

It is now recognized that neuropsychiatric symptoms (NPS), i.e., various disturbances of mood, perception, and behavior, are also a common manifestation of AD, emerging already before dementia onset [4]. In MCI, NPS have been associated with faster progression to dementia [5] and higher caregiver burden [6]. Their early and accurate detection is therefore highly clinically relevant.

The concept of mild behavioral impairment (MBI) has been developed to help identify NPS that may represent behavioral sequelae of preclinical AD and MCI due to AD. MBI is a neurobehavioral syndrome describing new-onset, persistent, and impactful NPS in older adults as a high-risk state for incident cognitive decline or progressing to dementia, which may be attributable to neurodegenerative processes [7]. The Mild Behavioral Impairment – Checklist (MBI-C) is an instrument created to detect MBI and assess the severity of a wide spectrum of NPS observed in this population [8], and it has been validated for the use in individuals with subjective cognitive decline and MCI [9–11].

Previous studies on NPS in preclinical AD and MCI due to AD often used instruments developed for assessing older adults with dementia, such as the Neuropsychiatric Inventory (NPI) [12, 13]. Although the NPI [14] is well-established and widely used, it might lack sufficient sensitivity to capture NPS in subjective cognitive decline or MCI populations. Other widely used NPS instruments include the Geriatric Depression Scale-15 item version (the GDS-15) [15] and the Beck Anxiety Inventory (BAI) [16], which are specific self-report instruments contingent on good comprehension and insight of the patients. A variety of NPS instruments used across studies may also account for inconsistency in relationships between NPS and AD biomarkers in the early stages of AD, highlighting the need for a more sensitive instrument [17, 18]. The MBI-C represents such an instrument to capture early, low-severity NPS typically found in populations at risk of dementia, which specifically operationalizes MBI criteria by stipulating symptoms are later-life emergent and persistent.

Studies on MBI in MCI from both community and clinical populations report symptoms of affective dysregulation, impulse dyscontrol, and apathy as the most common, while psychotic symptoms are rare [19, 20]. Although MBI in these studies was derived from the scores on the NPI, these findings were consistent with a later study using the MBI-C [10]. However, the etiology of the MCI was not specified in any of the studies. Therefore, while there is accumulating evidence supporting MBI as a manifestation of AD [21], the prevalence of MBI and the typical symptoms in biomarker-defined MCI due to AD remain to be elucidated.

Various causative mechanisms of NPS in AD have been proposed, including the neurodegenerative process, shared genetic and/or environmental risk factors or confounding, or a psychological reaction to perceived cognitive or functional decline [22]. Exploring their contribution is essential so that an appropriate treatment can be applied. There are several genetic risk factors that are known to influence the onset and progression of AD, but their role in NPS is still unclear.

Apolipoprotein (*APOE*) ɛ4 is an established genetic risk factor for late-onset AD [23, 24] and has been shown to influence not only the age of onset and rate of progression, but also the clinical phenotype [25–27]. Previous studies in AD dementia did not find consistent results regarding the role of *APOE* in NPS [28–30]. A recent systematic review and meta-analysis concluded that there was no association between *APOE* and the most common NPS in MCI and AD dementia; however, the vast majority of the participants had dementia, and the biomarker diagnoses were lacking [31]. On the other hand, evidence from cognitively healthy older adults suggests that *APOE* e4 carriage was associated with more severe self-reported depressive and anxiety symptoms irrespective of β-amyloid and more severe anxiety in β-amyloid positive individuals [32]. Regarding MBI, the evidence so far is scarce. One study reported an association of *APOE* e4 allele with affective dysregulation in non-demented older adults [33]. Another study found APOE to be an important moderator of progression to dementia among cognitively normal and MCI individuals with apathy domain [34]. Altogether, these studies suggest that the effect of *APOE* on NPS is possibly best captured before the dementia onset.

The brain-derived neurotrophic factor (*BDNF*) gene encodes the neurotrophin essential for neuronal growth, synaptogenesis, and experience-dependent synaptic plasticity [35]. The *BDNF* Val66Met is a risk gene polymorphism that adversely influences the clinical progression of AD [35], and it is also a risk factor for late-life depression [30, 36]. Despite these findings as well as the notion that late-life depression and depressive symptoms may represent a prodromal feature of AD [37–39], the association between *BDNF* and NPS has rarely been studied in the context of AD. Few studies found that the *BDNF* Met allele is found more often in individuals with AD dementia with depression compared to those without depression [40, 41]. Cognitively normal older women with *BDNF* Met reported more severe depressive symptoms compared to Val/Val carriers [32], which has been later observed to persist over time [42]. To our knowledge, no study has explored this risk in MCI due to AD. Moreover, the effect of *BDNF* Met is predominantly focused on depression, while the effect on other NPS remains understudied. One study found no associations between three *BDNF* polymorphism groups and NPS in amnestic MCI and AD dementia, although plasma BDNF level correlated with aggressiveness [43]. However, the study used Behave-AD, a measure developed to capture NPS occuring in the dementia stage.

Several studies have demonstrated a synergic deleterious effect of APOE e4 and BDNF Met genotypes on cognition [44, 45]. However, their synergic effect on the NPS is not yet known.

In our previous study, we reported an association between higher MBI-C severity and medial temporal lobe atrophy in a mixed sample of non-demented older adults with no biomarker evidence [46]. Building on our previous findings, in this study, we examined the association between the cognitive status of participants (amnestic MCI due to AD vs cognitively normal older adults) and the informant-rated MBI-C, the GDS-15, and the BAI. We expected that individuals with aMCI due to AD would have a higher severity of affective dysregulation, apathy, and impulse dyscontrol symptoms compared to cognitively normal older adults. Within aMCI due to AD, we further examined the potential influence of *APOE* and *BDNF* risk gene polymorphisms on MBI-C severity. We hypothesized that the presence of *APOE* and *BDNF* risk genetic polymorphisms would be associated with more severe MBI symptoms in aMCI due to AD individuals compared to non-carriers of these polymorphisms and that the interaction between *APOE* and/or *BDNF* polymorphisms would be significantly associated with MBI-C severity.

## Methods

### Participants

A total of 112 participants were recruited from the Czech Brain Aging Study, an ongoing longitudinal, observational, memory clinic-based study aimed at detecting early changes associated with pathological brain aging [47]; they were recruited from both Memory Clinics in Prague (*n* = 89) and Brno (*n* = 23). Participants with subjectively perceived cognitive complaints were referred to the Memory Clinic by general practitioners or other specialists. All participants underwent a standard diagnostic workup including neurological and laboratory evaluations, comprehensive neuropsychological examination, genotyping, and magnetic resonance imaging (MRI; 1.5 or 3 T with MP RAGE sequences). The cognitive status was established by cognitive neurologists and neuropsychologists based on clinical data, information provided by participants and their informants, and neuropsychological assessment (specified below). Individuals met the criteria for MCI according to NIA-AA 2011 criteria [2] based on (1) subjectively perceived cognitive decline compared to a previously normal status, (2) neuropsychologically confirmed objective cognitive impairment below 1.5 SD on at least two tests within a domain in at least one of five established cognitive domains, (3) preservation of independence in functional abilities (as confirmed in the clinical interviews), and (4) absence of dementia. All MCI participants underwent either lumbar puncture (*n* = 21), β-amyloid PET (*n* = 25), or both (*n* = 16) and had a positive AD biomarker signature based on either CSF and/or visual rating of a β-amyloid PET scan (see the procedures described below). In case of CSF/PET discordance (*n* = 1, due to a borderline β-amyloid level in CSF), the AD biomarker positivity was established using PET. Further, they had evidence of medial temporal lobe atrophy on MRI, rated visually by a trained cognitive neurologist. Only the amnestic type of MCI (aMCI), both single domain (*n* = 16) and multiple domain (*n* = 46), was included in the present study; we have decided not to include non-amnestic MCI participants due to their low number and atypical clinical profile (i.e., primary progressive aphasia or frontal variant of AD). This process resulted in including a total of 62 aMCI participants with a high likelihood of underlying AD pathology [2], further referenced as aMCI-AD.

Fifty cognitively normal older adults (CN) were recruited from the University of the Third Age or the patients’ relatives; they did not report significant subjective cognitive complaints for which they had ever sought medical attention; they had no evidence of brain atrophy or significant vascular changes (Fazekas > 2) on MRI, and their performance on neuropsychological testing was within normal limits. The demographic characteristics of all participants are presented in Table 1.

**Table 1:** Demographic, genetic, and neuropsychological characteristics of the participants

	**CN, *n* = 50, M ± SD**	**aMCI-AD, *n* = 62, M ± SD**
**Demographic characteristics**		
**Age**^a^	67.30 ± 6.50	72.34 ± 4.96^**^
**Female, *n* (%)**^b^	37 (74)	30 (48.4)^*^
**Education, years**^a^	16.12 ± 2.32	15.27 ± 2.89
**Genetic characteristics**		
***APOE* e4 + , *n* (%)**	–	45 (72.6)^†^
***BDNF* Met + , *n* (%)**	–	20 (32.3)^††^
**Neuropsychological characteristics**		
**MMSE, score**^a^	29.16 ± 0.87	25.48 ± 2.45^**^
**RAVLT 1–5, score**^a^	56.28 ± 7.72	32.52 ± 8.24^**^
**RAVLT delayed recall, score**^a^	11.84 ± 2.10	2.81 ± 3.03^**^
**LM delayed recall, score**^a^	17.48 ± 3.21	3.81 ± 4.31^**^
**ROCF recall, score**^a^	19.82 ± 6.30	6.70 ± 5.65^**^
**ROCF copy, score**^a^	31.09 ± 2.93	26.33 ± 6.86^**^
**Digit span, score**^a^	14.04 ± 4.49	14.03 ± 3.61
**TMT A, time to completion (s)**^a^	39.15 ± 12.59	57.50 ± 29.69^**^
**TMT B, time to completion (s)**^a^	77.86 ± 25.08	173.77 ± 84.76^**^
**PST**^a^	26.84 ± 6.40	47.42 ± 22.91^**^
**P-VF, score**^a^	51.14 ± 12.28	40.35 ± 11.86^**^
**C-VF animals, score**^a^	27.88 ± 5.61	18.48 ± 4.94^**^
**BNT-30, errors after a semantic cue**^a^	1.42 ± 2.00	4.65 ± 3.00^**^

*Abbreviations*: *CN* Cognitively normal older adults, *aMCI-AD* Amnestic mild cognitive impairment due to Alzheimer’s disease – high likelihood, *MMSE* Mini-Mental State Examination, *APOE* Apolipoprotein E, *BDNF* Brain-derived neurotrophic factor, *LM* Logical memory, *RAVLT* Rey Auditory Verbal Learning Test, *TMT* Trial Making Test, *BNT (30)* Boston Naming Test – 30-item version, *C-VF* Category verbal fluency, *P-VF* Phonemic verbal fluency – letters N, K, P, *PST* Prague Stroop Test, *ROCF* Rey-Osterrieth complex figure, *M* Mean, *SD* Standard deviation

^*^Difference from the CN group at *p* < 0.01

^**^Difference from the CN group at *p* < 0.001

^†^The information on *APOE* was missing for 1 participant. There were 37 e4 heterozygotes and 8 e4 homozygotes

^††^The information on *BDNF* was missing for 10 participants. There were 18 Met heterozygotes and 2 Met homozygotes

^a^*t*-test

^b^Chi-square

### Exclusion criteria

The exclusion criteria were (1) a diagnosis of dementia and (2) the presence of other neurologic or psychiatric disease (e.g., Parkinson’s disease, traumatic brain injury, stroke, alcohol or substance abuse, severe brain vascular burden (Fazekas > 2 on MRI), current major psychiatric disorder or a history of major psychiatric disorder as confirmed by clinical interviews).

### Neuropsychological assessment

The neuropsychological battery included the Mini-Mental State Examination (MMSE) as a screening of global cognitive function and the following tests to assess five cognitive domains [2]: (1) memory by the Rey Auditory Verbal Learning Test, Logical Memory from the Uniform Data Set, and Rey-Osterrieth Complex Figure Test (recall after 3 min); (2) executive function by the Trail Making Test B, phonemic verbal fluency—letters N, K, and P, and Prague Stroop test – colors; (3) language by the Boston Naming Test 30-item version and category verbal fluency – animals; (4) attention and working memory by the Trail Making Test A and Digit Span forward and backward from the Uniform Data Set; and (5) visuospatial function by the Rey-Osterrieth Complex Figure Test (copy) [48–51]. All scores are presented in Table 1.

### Neuropsychiatric assessment

The MBI-C, used in this study, is a 34-item rating scale designed to assess neuropsychiatric symptoms in older adults without dementia [8], which has been validated in individuals with subjective cognitive decline [11] and MCI [10]. It evaluates five behavioral domains in line with the MBI criteria: decreased motivation (apathy), affective dysregulation (mood/anxiety), impulse dyscontrol, social inappropriateness, and abnormal perception and thought content (psychotic symptoms). Each item is evaluated by the presence (yes/no) and severity of the symptoms (1, mild; 2, moderate; 3, severe).

The Czech version of the MBI-C [52] was completed by a participant’s close informant (a spouse/partner, a child, or another relative). A total score as well as five domain scores were calculated as a sum of the corresponding item severity ratings resulting in the *MBI-C total score* (0–102), *decreased motivation score* (0–18), *affective dysregulation score* (0–18), *impulse dyscontrol score* (0–36), *social inappropriateness score* (0–15), and *abnormal perception and thought content score* (0–15). *Z*-scores were calculated for the MBI-C total score and all the domain scores for the whole cohort. Participants with four or more missing items on the MBI-C were excluded. In case of three or fewer missing items, both total and domain scores were calculated without these items. The MBI diagnosis was based on the validated cutoffs of ≥ 9 and ≥ 7 for MBI in SCD and MCI, respectively [10, 11]. Across studies, other different cutoffs for MBI for cognitively normal or MCI have also been used, generally trending towards using a lower cutoff [9, 53–55]. To allow for comparison with such studies, we subsequently used a cutoff of ≥ 7 and an experimental cutoff of ≥ 6 for MBI for both groups for exploratory analysis of the MBI prevalence. In addition, two self-report instruments were administered to all the participants, the GDS-15 to measure depressive symptoms [15] and the BAI to measure anxiety symptoms [16]. Although these instruments are not specific to dementia, they have been commonly used in this population [56]. Based on these instruments, the *GDS-15 total score* (0–15) and *BAI total score* (0–66) were generated. The scores are presented in Table 2.

**Table 2:** Neuropsychiatric characteristics of the participants and the associations with cognitive status

Neuropsychiatric characteristics (range)			Unstandardized beta coefficients controlling for age and sex (95% CI)	*p*-value
	**CN, *n* = 50, M ± SD**	**aMCI-AD, *n* = 62, M ± SD**		
**MBI-C total (0–102)**	1.00 ± 1.82 (0–6)	8.13 ± 8.63 (0–43)	**3.33 (1.96–4.71)**	**< 0.001**
**MBI-C decreased motivation (0–18)**	0.20 ± 0.53 (0–3)	2.03 ± 2.79 (0–13)	**0.88 (0.44–1.32)**	**< 0.001**
**MBI-C affective dysregulation (0–18)**	0.27 ± 0.58 (0–2)	2.45 ± 2.62 (0–11)	**1.13 (0.71–1.54)**	**< 0.001**
**MBI-C impulse dyscontrol (0–36)**	0.48 ± 0.95 (0–3)	2.98 ± 3.59 (0–18)	**1.13 (0.55–1.70)**	**< 0.001**
**MBI-C social inappropriateness (0–15)**	0.08 ± 0.44 (0–3)	0.32 ± 0.72 (0–3)	0.10 (− 0.03–0.23)	0.123
**MBI-C abnormal perception/thought (0–15)**	0.06 ± 0.31 (0–2)	0.34 ± 1.13 (0–6)	0.10 (− 0.08–0.28)	0.290
**GDS-15 (0–15)**	1.36 ± 1.87 (0–8)	2.63 ± 2.07 (0–10)	**0.61 (0.19–1.03)**	**0.005**
**BAI (0–66)**	6.43 ± 5.99 (0–10)^a^	7.84 ± 7.25 (0–30)	0.81 (− 0.60–2.22)	0.256
**MBI diagnosis, *n* (%)**	0 (0)^b^ 3 (6)^d^	30 (48,4)^c^ 33 (53.2)^d^		

*Abbreviations*: *MBI-C* Mild behavioral impairment – checklist, *MBI* Mild behavioral impairment, *CN* Cognitively normal older adults, *aMCI-AD* Amnestic mild cognitive impairment due to Alzheimer’s disease – high likelihood, *GDS-15*, Geriatric Depression Scale-15 items, *BAI* Beck Anxiety Inventory, *CI* Confidence interval, *M* Mean, *SD* Standard deviation

^a^*n* = 49; for ne CN participant, the BAI was missing

^b^According to the validated cutoff ≥ 9 for MBI in SCD (Mallo et al., 2018b)

^c^According to the validated cutoff ≥ 7 for MBI in MCI (Mallo et al., 2018a)

^d^According to the experimental cutoff ≥ 6

### Genotyping

To determine the *APOE* genotype, DNA was isolated from blood samples (ethylenediaminetetraacetic acid; Qiagen extraction), and genotyping was performed according to Idaho-tech protocol (Luna Probes Genotyping Apolipoprotein [ApoE] Multiplexed Assay) for high-resolution melting analysis (HRM) [57, 58]. We developed an HRM analysis for the detection ofrs6265 (G196A) in the *BDNF* gene [44].

*APOE* and *BDNF* genotypes were available for 61 and 52 aMCI participants, respectively. The aMCI-AD participants were stratified based on the presence of at least one *APOE* and *BDNF* risk allele: for *APOE*, e4 heterozygotes (*n* = 37) and homozygotes (*n* = 8) were pooled into a group of e4 carriers (e4 + ; *n* = 45), and the rest of the participants represented non-carriers (e4 − ; *n* = 16). Participant with aMCI-AD carrying *APOE* e2 allele (*n* = 1) was not included. Similarly, for *BDNF*, Met heterozygotes (*n* = 18) and homozygotes (*n* = 2) were pooled into a group of Met carriers (Met + ; *n* = 20), and the rest of the participants represented non-carriers (Met − ; *n* = 32).

### Cerebrospinal fluid analysis of AD biomarkers

The CSF samples were obtained by lumbar puncture with an atraumatic needle in the lying position. The first 3 ml of CSF was used for routine analysis, and the remaining 10 ml of CSF was centrifuged and stored at − 80 °C 30 min after the puncture. CSF collection, processing, and archiving were performed in accordance with European recommendations [59]. β-Amyloid_1–42_ in CSF was analyzed using a commercial ELISA kit (Euroimmun) in the Cerebrospinal Fluid Laboratory, Institute of Immunology and Department of Neurology, Second Faculty of Medicine, Charles University and Motol University Hospital.

### Amyloid PET imaging

The PET images were acquired using a Biograph 40 TrueV HD PET/CT scanner (Siemens Healthineers AG, Erlangen, Germany) in the Department of Nuclear Medicine and PET Centre, Na Homolce Hospital. The participants received a single intravenous dose of flutemetamol (18F; Vizamyl, GE Healthcare, Chicago, IL). Non-contrast low-dose CT brain images were acquired for attenuation correction prior to the PET scans. A PET list-mode acquisition was performed in two phases: early (perfusion) and late (β-amyloid). The early-phase images were acquired at the time of flutemetamol (18F) administration for 8 min and rebinned into dynamic datasets of 2 × 4 min for motion checking. The late-phase images were acquired 90 min after flutemetamol (18F) administration for a total of 10 min (2 × 5 min). Flutemetamol (18F) PET images were visually read (as positive or negative) by a certified nuclear medicine specialist using the GM-EDGE method [60].

### Statistical analysis

Descriptive statistics were provided to characterize the sample using means and standard deviations (Table 1). We used *t*-tests and chi-square tests to explore the between-group differences in demographic and neuropsychological characteristics. We then estimated the association between our main independent variable—cognitive status (aMCI-AD or CN), and one of our outcomes—the MBI-C total score, MBI-C subdomains, GDS-15, and BAI—using multiple linear regression, controlling for the effects of age and sex (Table 2). Because the groups did not differ in years of education, education was not considered as a covariate in these analyses.

Next, we restricted the sample to aMCI-AD only and examined *APOE* e4 + and *BDNF* Met + in relation to MBI-C total score (Additional file 1: Table S1, Model 1.1, 1.2), MBI-C domain scores (Additional file 1: Tables S2.1-S2.3), GDS-15 (Additional file 1: Table S3, Model 3.1, 3.2), and BAI (Additional file 1: Table S4, Model 4.1, 4.2) using a multiple linear regression controlling for age, sex, and MMSE score. Then, we added an interaction term (of *APOE-by-BDNF*) to these models (models 1.3, 2.3.1–2.3.5, 3.3, and 4.3).

For detailed results of the genetic analyses, see Additional file 1: Tables S1-S4 published as supplementary material.

## Results

The demographic, neuropsychological, and neuropsychiatric characteristics of the CN (*n* = 50) and aMCI-AD (*n* = 62) groups are presented in Table 1. All participants were Caucasian. The aMCI-AD individuals were older, and there were fewer women compared to CN individuals. The groups did not differ in years of education. In terms of their cognitive functioning, as expected, aMCI-AD performed worse in all the neuropsychological tests except the Digit Span. Among AD aMCI participants, 15 (24%) were taking antidepressants (SSRI) and 23 (21%) were taking cognitive-enhancing medication (cholinesterase inhibitors: *n* = 22; memantine: *n* = 1; their combination: *n* = 1).

Overall, 28% (14/50 individuals) of the CN and 82% (51/62 individuals) of the aMCI-AD group had an MBI-C total score > 0. No one from the CN group met the criteria for MBI (defined as MBI-C total score ≥ 9 based on the validated cutoff for SCD [11]); on the other hand, 48.4% (i.e., 31/62 individuals) of the aMCI-AD group met the criteria for MBI (defined as MBI-C total score ≥ 7 based on the validated cutoff for MCI [10]). Using the cutoff of ≥ 7 for both groups did not change the proportion of MBI in the CN; however, when a lower cutoff of ≥ 6 was applied for both groups, 6% (*n* = 3) of the CN and 53.2% (*n* = 33) of the aMCI-AD individuals met the criteria for MBI.

The results for the association between cognitive status and MBI outcomes are presented in Table 2. Having aMCI-AD was associated with a 3.33 point higher overall MBI severity (*B* = 3.33 [1.96, 4.71], *p* < 0.001) than being cognitively healthy. Specifically, aMCI-AD was associated with higher affective dysregulation (*B* = 1.13 [0.71, 1.54], *p* < 0.001), apathy (*B* = 0.88 [0.44, 1.32], *p* < 0.001), and impulse dyscontrol symptoms (*B* = 1.13 [0.55, 1.70], *p* < 0.001). Cognitive status was not related to social inappropriateness or psychotic symptoms (*p* > 0.05). Regarding self-rated NPS instruments, we found that aMCI-AD was associated with a 0.61-point higher severity of depression (*B* = 0.61 [0.19, 1.03], *p* = 0.005) compared to being cognitively healthy, but there was no association with anxiety severity (based on the GDS-15 and BAI scores, respectively).

The results for the association between genetic polymorphisms (i.e., APOE and BDNF) and NPS outcomes (i.e., MBI-C total and domain scores, GDS-15, and BAI) are presented in Additional file 1: Tables S1-S4. Regarding the association between genetic polymorphisms and MBI severity, we found that neither the *APOE* e4 + nor *BDNF* Met + were related to the overall MBI severity in separate regression models (*B* = 0.52 [− 4.78–5.82], *p* = 0.846 and *B* =  − 1.29 [− 6.92–4.34], *p* = 0.646, respectively). Additionally, when entered into the same regression model, neither genetic polymorphism was related to MBI severity. The interaction of both genetic polymorphisms did not relate significantly to MBI-C total score (*B* =  − 0.40 [− 11.89–12.69], *p* = 0.948) or domain scores (see Additional file 1: Tables S1 and S2). Similar results were obtained for the GDS-15 score (*p* > 0.05) (see Additional file 1: Table S3).

We observed no association between *APOE* e4 + (*B* =  − 3.52 [− 7.84–0.80], *p* = 0.108) or *BDNF* Met + (*B* = 3.78 [− 0.84–8.40], *p* = 0.107) and the BAI score and a borderline non-significant association between APOE*BDNF interaction (*B* =  − 9.29 [− 18.78–0.20], *p* = 0.055) and the BAI score. However, our results suggested that Met + was related to a higher BAI score than e4 − /Met − after controlling for age, sex, MMSE score, the independent effect of *APOE* e4 + , and the APOE*BDNF interaction (*B* = 10.73 [2.51, 18.94], *p* = 0.012). This model also offered the best fit (Model 4.3 in the Additional file 1: Table S4).

## Discussion

The main findings of the present study using the informant-rated MBI-C and two self-rated NPS instruments are as follows: (1) aMCI due to AD was associated with more severe symptoms of apathy, affective dysregulation, and impulse dyscontrol but not socially inappropriate behavior or psychotic symptoms compared to healthy controls; (2) almost half of the aMCI due to AD individuals fulfilled the criteria for MBI syndrome; (3) neither the presence of *APOE* e4 nor *BDNF* Met was related to MBI symptom severity in aMCI due to AD individuals; and (4) there appears to be a positive association between BDNF Met carriership and BAI score.

Our findings thus complement recent evidence of MBI in preclinical AD and its association with AD biomarkers [61–63] by examining NPS in MCI due to AD and suggest that its severity is not related to the presence of *APOE* e4 or *BDNF* Met allele.

Almost half of the aMCI-AD participants fulfilled the criteria for the MBI syndrome (as defined by the validated MBI-C cutoff ≥ 7) [10]. Very few studies to date examined the MBI with the MBI-C in a memory clinic sample, but our results are similar to the findings from previous specialized memory clinic MCI samples from Canada [9] and Iran [64]. The substantially higher prevalence compared to a previous Spanish study [10] is likely explained by different, more conservative criteria used for MBI ascertainment in that study, and the fact that participants were from primary care rather than specialist clinics. Using a lower cutoff ≥ 6 resulted in a slight increase in the MBI prevalence both in the aMCI-AD and CN groups (6% of the individuals of the latter met the MBI criteria). Evidence shows that MBI can precede MCI, although with a variable prevalence due to heterogeneity in methodology [65]. Thus, observing MBI in our CN group is not surprising. In fact, these individuals could be at higher risk of developing MCI as previous studies have shown that MBI was related to AD pathology in CN older adults [62, 63] and that stratification of CN older adults based on this cutoff strengthened the association between genetic risk for AD and poorer cognitive performance [55].

Based on our findings, having aMCI-AD was associated with greater apathy, affective dysregulation, and impulse dyscontrol compared to being cognitively normal, which is in accordance with previous studies using both MBI-C [9, 10] and NPI to diagnose MBI [19, 20]. A number of other studies on NPS also support these findings [4, 5, 66]. However, MCI in these studies was defined using only clinical criteria without biomarker evidence. One recent study assessed β-amyloid-positive individuals across the cognitive spectrum with the NPI and consistent with our findings reported apathy, irritability, depression, and anxiety as the four most common NPS in MCI [12]. The literature also highlights that MCI individuals with these NPS are at a higher risk of progression to AD dementia [5, 67], while fewer affective symptoms and their improvement were associated with a higher likelihood of reversion from MCI to normal cognition [68]. Taken together, these symptoms are the most common (although not specific) neuropsychiatric manifestation in MCI due to AD, and their early and accurate identification helps recognize individuals at higher risk of progression to dementia.

There was no association between cognitive status and socially inappropriate behavior or psychotic symptoms, and the mean MBI-C scores in these domains were very low on average. Socially inappropriate behavior is typical for the early stages of frontotemporal dementia [69], and psychotic symptoms are common in Lewy body disease, even in its prodromal stages [70]. In AD, these symptoms usually emerge in the dementia stage. However, on an individual level, even subthreshold symptoms in these domains may be clinically very important. Especially, the presence of psychotic symptoms in MCI is associated with a higher risk of progression to dementia [71] and according to a recent study represented the highest risk factor for progression among all NPS [72]. Thus, these symptoms should still be assessed in MCI participants regardless of their prevalence.

Using the traditional self-rated NPS measures, we found that aMCI-AD was associated with reporting more severe depressive symptoms compared to healthy controls, even though these symptoms were mostly subthreshold. These results are consistent with previous studies and support that these symptoms are among the earliest and most frequently reported NPS in MCI [73, 74]. Notably, in some studies, individuals with GDS-15 ≥ 6 (indicating mild depressive symptoms), are excluded, and it is also among the exclusion criteria for the Alzheimer’s Disease Neuroimaging Initiative database at baseline. In the current study, we did not exclude participants based on the GDS-15 score; however, only two (3%) aMCI-AD scored above this threshold, and no participant scored ≥ 11 (indicating severe depressive symptoms).

There was no association between cognitive status and healthy self-reported anxiety symptoms, as measured by the BAI. All individuals were free from any prior or concurrent formal psychiatric disorder, but the average BAI scores were in the range of minimal to mild anxiety symptoms for both groups. This finding may seem inconsistent when compared to the MBI-C affective dysregulation, where the association was notable. However, the MBI-C affective dysregulation is informant-rated, includes both dysphoria and anxiety, and requires a 6-month symptom duration. On the contrary, the BAI is self-report and requires a 1-month symptom duration. It is also possible that in some CN older adults, slightly higher anxiety symptoms indicated by BAI scores may be more often connected to recent challenges or stressful life events, and/or these symptoms may remain unnoticed by the informants.

Our results do not support associations between *APOE* e4 or *BDNF* Met allele and MBI severity in aMCI-AD, even with the use of the MBI-C as a measure sensitive to NPS in the predementia population. One previous study showed that *APOE* e4 carriers had an increased likelihood of affective dysregulation measured by the MBI-C [33]. The study included a mixed sample of > 1200 cognitively heterogeneous non-demented participants without AD biomarkers. Thus, a higher severity of NPS could be explained by a presumably higher number of individuals with preclinical or prodromal AD in their *APOE* e4-positive group. In such case, the differences would not be a direct consequence of the *APOE* e4 positivity.

Both *APOE* and *BDNF* risk polymorphisms have been linked to a higher risk of late-life depression in non-demented older adults [30]. These studies did not include AD biomarkers either. Late-life depression could represent an early symptom of AD, which could moderate the relationship with the polymorphisms. Using AD biomarkers in our study has already increased the probability of underlying AD. As a result, these polymorphisms may have weaker associations with NPS severity. In fact, previous research also cautions about the positive associations specifically with the *APOE* [30], as it has been found that the association between *APOE* and late-life depression in a combined sample of healthy older adults and AD dementia did not survive stratification by the presence of AD dementia [75].

Our negative findings may also be influenced by sample characteristics. Most previous studies were performed in individuals with dementia [28, 30], where NPS are generally more prevalent and severe. Another issue could be a substantially higher prevalence of *APOE* e4 + compared to e4−in our aMCI-AD sample (45 (72.6%) vs 16 (25.8%) individuals, respectively), which is not surprising given that the *APOE* e4 allele is the strongest genetic risk factor for late-onset AD [24].

Additionally, the relationship between genetic polymorphisms and NPS could be moderated by other factors. For example, sex has been suggested to moderate the relationship between APOE and BDNF genotype and NPS in healthy older adults [32] and APOE and NPS in clinical cohorts [76]. Our analyses were controlled for the effect of sex. However, examining sex-specific associations could be the aim of future studies.

Contrary to the negative findings when examined separately, there seems to be an association between the *BDNF* Met risk allele and self-reported anxiety when controlling for the *APOE* e4 + and APOE*BDNF interactive effects. The cumulative effect of these polymorphisms (i.e., the co-occurrence of *APOE* e4 + and *BDNF* Met +) has been previously found to negatively influence memory and spatial navigation in aMCI individuals [44, 45]. However, prior research on NPS has mostly focused on these polymorphisms separately. Our study shows that the *BDNF* Met allele influenced self-reported anxiety when the presence of *APOE* e4 and the interaction with *APOE* e4 was considered. In agreement with previous conclusions [31], our results support examining the effect of multiple genes on NPS instead of focusing on individual polymorphisms. These results need to be replicated in larger samples.

Although the current study made several contributions on the topic of *APOE* and *BDNF* genetic polymorphisms and MBI in those with aMCI, the *R*^2^ values were modest across all models. This suggests that a large portion of the variance in MBI-C, GDS, and BAI was left unexplained. The effect sizes of *APOE* and *BDNF* genetic polymorphisms on MBI may be small when compared to other biological, psychological, or environmental factors, which have been suggested as possible mechanisms linking NPS with AD [22].

It is however important to note that the previously observed tendency of NPS to change over time [12] poses an inherent challenge for cross-sectional designs to capture associations with genetic polymorphisms. Longitudinal studies could thus provide a better insight. A recent study of a mixed sample of > 3900 CN and MCI individuals with no biomarkers found that apathy (regardless of other NPS) contributed to a higher risk of developing dementia in *APOE* e3 carriers compared to e4 carriers [34]. Therefore, the effect of genetic polymorphisms could manifest rather as a modifying factor of NPS trajectory, which is a hypothesis worth exploring further.

To our knowledge, this is the first study using the MBI-C to characterize MBI in a sample of neuropsychologically well-defined aMCI due to AD with biomarker evidence. However, we acknowledge several limitations to our study. First, the memory clinic setting limits the generalizability of our results, since NPS are more prevalent in clinic-based compared to community-based samples [73]. Also, a small sample size could have decreased the statistical power to detect the effects of the genetic polymorphism groups. We did not control for multiple comparisons, as we think that the study is already biased towards type II error in that the sample is particularly small (and unique). The associations suggested here should be therefore verified in larger samples. Due to a small number of homozygotes in our study (eight *APOE* e4 and two *BDNF* Met), we were not able to examine a dose-dependent effect of the polymorphisms on MBI. Furthermore, we lacked information about the biomarker profile of CN individuals; therefore, some of them may have already been in the preclinical stage of AD. However, all CN participants were carefully selected based on their medical history, they had no cognitive complaints, and their normal cognitive status was confirmed by an extensive neuropsychological evaluation. Comparing the MBI profile between preclinical and prodromal AD (i.e., MCI) would undoubtedly be an interesting aim of future studies. In addition to the MBI-C, we used two widely used self-report NPS instruments (i.e., the GDS-15 and the BAI). However, we acknowledge that a comparison with other informant-report measures, such as the NPI-Q [77] would be of great interest.

## Conclusions

In conclusion, aMCI due to AD was associated with more severe affective dysregulation, apathy, and impulse dyscontrol symptoms than cognitively healthy older adults, and MBI was present in almost half of the aMCI-AD participants. Neither the presence of *APOE* e4 nor *BDNF* was associated with the severity of the NPS when considered separately. However, their combined effect on NPS warrants further investigation, especially in larger samples and with biomarker status in the CN group.

### Supplementary Information

**Additional file 1:** **Table S1.** The association between MBI total score and APOE and BDNF polymorphism groups. **Table S2.1.** The association between MBI domain scores and APOE polymorphism groups. **Table S2.2.** The association between MBI domain scores and BDNF polymorphism groups. **Table S2.3.** The association between MBI domain scores and APOE and BDNF polymorphism groups. **Table S3.** The association between GDS-15 and APOE and BDNF polymorphism groups. **Table S4.** The association between BAI and APOE and BDNF polymorphism groups.

## Data Availability

The datasets used and/or analyzed during the current study are available from the corresponding author upon reasonable request. The MBI-C can be obtained at https://mbitest.org/.

## Abbreviations

AD: Alzheimer’s disease
AD-aMCI: Amnestic mild cognitive impairment due to Alzheimer’s disease (high likelihood)
aMCI: Amnestic mild cognitive impairment
APOE: Apolipoprotein E
BAI: Beck Anxiety Inventory
BDNF: Brain-derived neurotrophic factor
CN: Cognitively normal older adults
GDS-15: Geriatric Depression Scale-15-item version
MBI: Mild behavioral impairment
MBI-C: Mild Behavioral Impairment – Checklist
MCI: Mild cognitive impairment
NPI: Neuropsychiatric Inventory
NPS: Neuropsychiatric symptoms

## References

[1] McKhann GM, Knopman DS, Chertkow H, Hyman BT, Jack CR, Kawas CH (2011). The diagnosis of dementia due to Alzheimer’s disease: recommendations from the National Institute on Aging-Alzheimer’s Association workgroups on diagnostic guidelines for Alzheimer’s disease. Alzheimers Dement.

[2] Albert MS, DeKosky ST, Dickson D, Dubois B, Feldman HH, Fox NC (2011). The diagnosis of mild cognitive impairment due to Alzheimer’s disease: recommendations from the National Institute on Aging-Alzheimer’s Association workgroups on diagnostic guidelines for Alzheimer’s disease. Alzheimers Dement.

[3] Jack CR, Bennett DA, Blennow K, Carrillo MC, Dunn B, Haeberlein SB (2018). NIA-AA Research Framework: toward a biological definition of Alzheimer’s disease. Alzheimers Dement.

[4] Wise EA, Rosenberg PB, Lyketsos CG, Leoutsakos JM (2019). Time course of neuropsychiatric symptoms and cognitive diagnosis in National Alzheimer’s Coordinating Centers volunteers. Alzheimers Dement (Amst).

[5] Roberto N, Portella MJ, Marquié M, Alegret M, Hernández I, Mauleón A (2021). Neuropsychiatric profiles and conversion to dementia in mild cognitive impairment, a latent class analysis. Sci Rep.

[6] Paradise M, McCade D, Hickie IB, Diamond K, Lewis SJG, Naismith SL (2015). Caregiver burden in mild cognitive impairment. Aging Ment Health.

[7] Ismail Z, Smith EE, Geda Y, Sultzer D, Brodaty H, Smith G (2016). Neuropsychiatric symptoms as early manifestations of emergent dementia: provisional diagnostic criteria for mild behavioral impairment. Alzheimers Dement.

[8] Ismail Z, Agüera-Ortiz L, Brodaty H, Cieslak A, Cummings J, Fischer CE (2017). The Mild Behavioral Impairment Checklist (MBI-C): a rating scale for neuropsychiatric symptoms in pre-dementia populations. J Alzheimers Dis.

[9] Hu S, Patten S, Charlton A, Fischer K, Fick G, Smith EE (2022). Validating the mild behavioral impairment checklist in a cognitive clinic: comparisons with the Neuropsychiatric Inventory Questionnaire. J Geriatr Psychiatry Neurol.

[10] Mallo SC, Ismail Z, Pereiro AX, Facal D, Lojo-Seoane C, Campos-Magdaleno M (2018). Assessing mild behavioral impairment with the Mild Behavioral Impairment-Checklist in people with mild cognitive impairment. J Alzheimers Dis.

[11] Mallo SC, Ismail Z, Pereiro AX, Facal D, Lojo-Seoane C, Campos-Magdaleno M (2018). Assessing mild behavioral impairment with the mild behavioral impairment checklist in people with subjective cognitive decline. Int Psychogeriatr.

[12] Eikelboom WS, van den Berg E, Singleton EH, Baart SJ, Coesmans M, Leeuwis AE (2021). Neuropsychiatric and cognitive symptoms across the Alzheimer disease clinical spectrum: cross-sectional and longitudinal associations. Neurology.

[13] Wang Y, Lou F, Li Y, Liu F, Wang Y, Cai L (2021). Clinical, neuropsychological, and neuroimaging characteristics of amyloid-positive vs. amyloid-negative patients with clinically diagnosed Alzheimer’s disease and amnestic mild cognitive impairment. Curr Alzheimer Res.

[14] Cummings JL (1997). The Neuropsychiatric Inventory: assessing psychopathology in dementia patients. Neurology.

[15] Sheikh JI, Yesavage JA (1986). Geriatric Depression Scale (GDS): recent evidence and development of a shorter version. Clin Gerontol.

[16] Beck AT, Epstein N, Brown G, Steer RA (1988). An inventory for measuring clinical anxiety: psychometric properties. J Consult Clin Psychol.

[17] Banning LCP, Ramakers IHGB, Deckers K, Verhey FRJ, Aalten P (2019). Affective symptoms and AT(N) biomarkers in mild cognitive impairment and Alzheimer’s disease: a systematic literature review. Neurosci Biobehav Rev.

[18] Ng KP, Chiew H, Rosa-Neto P, Kandiah N, Ismail Z, Gauthier S (2021). Associations of AT(N) biomarkers with neuropsychiatric symptoms in preclinical Alzheimer’s disease and cognitively unimpaired individuals. Transl Neurodegener.

[19] Mortby ME, Ismail Z, Anstey KJ (2018). Prevalence estimates of mild behavioral impairment in a population-based sample of pre-dementia states and cognitively healthy older adults. Int Psychogeriatr.

[20] Sheikh F, Ismail Z, Mortby ME, Barber P, Cieslak A, Fischer K (2018). Prevalence of mild behavioral impairment in mild cognitive impairment and subjective cognitive decline, and its association with caregiver burden. Int Psychogeriatr.

[21] Creese B, Ismail Z (2022). Mild behavioral impairment: measurement and clinical correlates of a novel marker of preclinical Alzheimer’s disease. Alzheimers Res Ther.

[22] Geda YE, Schneider LS, Gitlin LN, Miller DS, Smith GS, Bell J (2013). Neuropsychiatric symptoms in Alzheimer’s disease: past progress and anticipation of the future. Alzheimers Dement.

[23] Liu CC, Liu CC, Kanekiyo T, Xu H, Bu G (2013). Apolipoprotein E and Alzheimer disease: risk, mechanisms and therapy. Nat Rev Neurol.

[24] Roses AD (1996). Apolipoprotein E alleles as risk factors in Alzheimer’s disease. Annu Rev Med.

[25] Joie RL, Visani AV, Lesman-Segev OH, Baker SL, Edwards L, Iaccarino L (2021). Association of APOE4 and clinical variability in Alzheimer disease with the pattern of tau- and amyloid-PET. Neurology.

[26] Scheltens NME, Tijms BM, Koene T, Barkhof F, Teunissen CE, Wolfsgruber S (2017). Cognitive subtypes of probable Alzheimer’s disease robustly identified in four cohorts. Alzheimers Dement.

[27] Weintraub S, Teylan M, Rader B, Chan KCG, Bollenbeck M, Kukull WA (2020). APOE is a correlate of phenotypic heterogeneity in Alzheimer disease in a national cohort. Neurology.

[28] Borroni B, Costanzi C, Padovani A (2010). Genetic susceptibility to behavioural and psychological symptoms in Alzheimer disease. Curr Alzheimer Res.

[29] Panza F, Frisardi V, Seripa D, D’Onofrio G, Santamato A, Masullo C (2012). Apolipoprotein E genotypes and neuropsychiatric symptoms and syndromes in late-onset Alzheimer’s disease. Ageing Res Rev.

[30] Tsang RSM, Mather KA, Sachdev PS, Reppermund S (2017). Systematic review and meta-analysis of genetic studies of late-life depression. Neurosci Biobehav Rev.

[31] Banning LCP, Ramakers IHGB, Deckers K, Verhey FRJ, Aalten P (2019). Apolipoprotein E and affective symptoms in mild cognitive impairment and Alzheimer’s disease dementia: a systematic review and meta-analysis. Neurosci Biobehav Rev.

[32] Holmes SE, Esterlis I, Mazure CM, Lim YY, Ames D, Rainey-Smith S (2016). β-Amyloid, APOE and BDNF genotype, and depressive and anxiety symptoms in cognitively normal older women and men. Am J Geriatr Psychiatry.

[33] Andrews SJ, Ismail Z, Anstey KJ, Mortby M (2018). Association of Alzheimer’s genetic loci with mild behavioral impairment. Am J Med Genet B Neuropsychiatr Genet.

[34] Vellone D, Ghahremani M, Goodarzi Z, Forkert ND, Smith EE, Ismail Z (2022). Apathy and APOE in mild behavioral impairment, and risk for incident dementia. Alzheimers Dement (N Y).

[35] Brown DT, Vickers JC, Stuart KE, Cechova K, Ward DD (2020). The BDNF Val66Met polymorphism modulates resilience of neurological functioning to brain ageing and dementia: a narrative review. Brain Sci.

[36] Pei Y, Smith AK, Wang Y, Pan Y, Yang J, Chen Q (2012). The brain-derived neurotrophic-factor (BDNF) val66met polymorphism is associated with geriatric depression: a meta-analysis. Am J Med Genet B Neuropsychiatr Genet.

[37] Singh-Manoux A, Dugravot A, Fournier A, Abell J, Ebmeier K, Kivimäki M (2017). Trajectories of depressive symptoms before diagnosis of dementia. JAMA Psychiat.

[38] Steenland K, Karnes C, Seals R, Carnevale C, Hermida A, Levey A (2012). Late-life depression as a risk factor for mild cognitive impairment or Alzheimer’s disease in 30 US Alzheimer’s disease centers. J Alzheimers Dis.

[39] Tapiainen V, Hartikainen S, Taipale H, Tiihonen J, Tolppanen AM (2017). Hospital-treated mental and behavioral disorders and risk of Alzheimer’s disease: a nationwide nested case-control study. Eur Psychiatry.

[40] Borroni B, Archetti S, Costanzi C, Grassi M, Ferrari M, Radeghieri A (2009). Role of BDNF Val66Met functional polymorphism in Alzheimer’s disease-related depression. Neurobiol Aging.

[41] Zhang L, Fang Y, Zeng Z, Lian Y, Wei J, Zhu H (2011). BDNF gene polymorphisms are associated with Alzheimer’s disease-related depression and antidepressant response. J Alzheimers Dis.

[42] Holmes SE, Esterlis I, Mazure CM, Lim YY, Ames D, Rainey-Smith S (2018). Trajectories of depressive and anxiety symptoms in older adults: a 6-year prospective cohort study. Int J Geriatr Psychiatry.

[43] Nagata T, Kobayashi N, Shinagawa S, Yamada H, Kondo K, Nakayama K (2014). Plasma BDNF levels are correlated with aggressiveness in patients with amnestic mild cognitive impairment or Alzheimer disease. J Neural Transm.

[44] Cechova K, Andel R, Angelucci F, Chmatalova Z, Markova H, Laczó J (2020). Impact of APOE and BDNF Val66Met gene polymorphisms on cognitive functions in patients with amnestic mild cognitive impairment. J Alzheimers Dis.

[45] Laczó J, Cechova K, Parizkova M, Lerch O, Andel R, Matoska V (2020). The combined effect of APOE and BDNF Val66Met polymorphisms on spatial navigation in older adults. J Alzheimers Dis.

[46] Matuskova V, Ismail Z, Nikolai T, Markova H, Cechova K, Nedelska Z (2021). Mild behavioral impairment is associated with atrophy of entorhinal cortex and hippocampus in a memory clinic cohort. Front Aging Neurosci.

[47] Sheardova K, Vyhnalek M, Nedelska Z, Laczo J, Andel R, Marciniak R (2019). Czech Brain Aging Study (CBAS): prospective multicentre cohort study on risk and protective factors for dementia in the Czech Republic. BMJ Open.

[48] Nikolai T, Stepankova H, Kopecek M, Sulc Z, Vyhnalek M, Bezdicek O (2018). The uniform data set, Czech version: normative data in older adults from an international perspective. J Alzheimers Dis.

[49] Nikolai T, Štěpánková H, Michalec J, Bezdíček O, Horáková K, Marková H (2015). Verbal Fluency Tests – Czech normative study for older persons. Cesk Slov Neurol N.

[50] Drozdová K, Štěpánková H, Lukavský J, Bezdíček O, Kopeček M (2015). Normative data for the Rey- Osterrieth Complex Figure Test in older Czech adults. Cesk Slov Neurol N.

[51] Bezdicek O, Lukavsky J, Stepankova H, Nikolai T, Axelrod BN, Michalec J (2015). The Prague Stroop Test: normative standards in older Czech adults and discriminative validity for mild cognitive impairment in Parkinson’s disease. J Clin Exp Neuropsychol.

[52] Matuskova V, Nikolai T, Markova H, Cechova K, Laczo J, Hort J (2020). Neuropsychiatrické symptomy jako časná manifestace Alzheimerovy nemoci. Cesk Slov Neurol N.

[53] Kassam F, Chen H, Nosheny RL, McGirr A, Williams T, Ng N (2022). Cognitive profile of people with mild behavioral impairment in Brain Health Registry participants. Int Psychogeriatr.

[54] Ghahremani M, Nathan S, Smith EE, McGirr A, Goodyear B, Ismail Z (2023). Functional connectivity and mild behavioral impairment in dementia-free elderly. Alzheimers Dement.

[55] Creese B, Arathimos R, Brooker H, Aarsland D, Corbett A, Lewis C (2021). Genetic risk for Alzheimer’s disease, cognition, and mild behavioral impairment in healthy older adults. Alzheimers Dement.

[56] Gitlin LN, Marx KA, Stanley IH, Hansen BR, Van Haitsma KS (2014). Assessing neuropsychiatric symptoms in people with dementia: a systematic review of measures. Int Psychogeriatr.

[57] Hixson JE, Vernier DT (1990). Restriction isotyping of human apolipoprotein E by gene amplification and cleavage with HhaI. J Lipid Res.

[58] Laczó J, Andel R, Vlček K, Macoška V, Vyhnálek M, Tolar M (2011). Spatial navigation and APOE in amnestic mild cognitive impairment. Neurodegener Dis.

[59] Vanderstichele H, Bibl M, Engelborghs S, Le Bastard N, Lewczuk P, Molinuevo JL (2012). Standardization of preanalytical aspects of cerebrospinal fluid biomarker testing for Alzheimer’s disease diagnosis: a consensus paper from the Alzheimer’s Biomarkers Standardization Initiative. Alzheimers Dement.

[60] Belohlavek O, Jaruskova M, Skopalova M, Szarazova G, Simonova K (2019). Improved beta-amyloid PET reproducibility using two-phase acquisition and grey matter delineation. Eur J Nucl Med Mol Imaging.

[61] Ghahremani M, Wang M, Chen HY, Zetterberg H, Smith E, Ismail Z (2023). Plasma phosphorylated tau at threonine 181 and neuropsychiatric symptoms in preclinical and prodromal Alzheimer disease. Neurology.

[62] Lussier FZ, Pascoal TA, Chamoun M, Therriault J, Tissot C, Savard M (2020). Mild behavioral impairment is associated with β-amyloid but not tau or neurodegeneration in cognitively intact elderly individuals. Alzheimers Dement.

[63] Johansson M, Stomrud E, Insel PS, Leuzy A, Johansson PM, Smith R (2021). Mild behavioral impairment and its relation to tau pathology in preclinical Alzheimer’s disease. Transl Psychiatry.

[64] Kianimehr G, Fatehi F, Noroozian M. Prevalence of mild behavioral impairment in patients with mild cognitive impairment. Acta Neurol Belg. 2022;122:1493–7.10.1007/s13760-021-01724-z. 10.1007/s13760-021-01724-z 34191260

[65] Pan Y, Shea YF, Li S, Chen R, Mak HKF, Chiu PKC (2021). Prevalence of mild behavioural impairment: a systematic review and meta-analysis. Psychogeriatrics.

[66] Geda YE, Roberts RO, Knopman DS, Petersen RC, Christianson TJH, Pankratz VS (2008). Prevalence of neuropsychiatric symptoms in mild cognitive impairment and normal cognitive aging: population-based study. Arch Gen Psychiatry.

[67] Rosenberg PB, Mielke MM, Appleby BS, Oh ES, Geda YE, Lyketsos CG (2013). The association of neuropsychiatric symptoms in MCI with incident dementia and Alzheimer disease. Am J Geriatr Psychiatry.

[68] Sugarman MA, Alosco ML, Tripodis Y, Steinberg EG, Stern RA (2018). Neuropsychiatric symptoms and the diagnostic stability of mild cognitive impairment. J Alzheimers Dis.

[69] Desmarais P, Lanctôt KL, Masellis M, Black SE, Herrmann N (2018). Social inappropriateness in neurodegenerative disorders. Int Psychogeriatr.

[70] Fischer CE, Agüera-Ortiz L (2018). Psychosis and dementia: risk factor, prodrome, or cause?. Int Psychogeriatr.

[71] Ismail Z, Creese B, Aarsland D, Kales HC, Lyketsos CG, Sweet RA (2022). Psychosis in Alzheimer disease—mechanisms, genetics and therapeutic opportunities. Nat Rev Neurol.

[72] Dietlin S, Soto M, Kiyasova V, Pueyo M, de Mauleon A, Delrieu J (2019). Neuropsychiatric symptoms and risk of progression to Alzheimer’s disease among mild cognitive impairment subjects. J Alzheimers Dis.

[73] Ismail Z, Elbayoumi H, Fischer CE, Hogan DB, Millikin CP, Schweizer T (2017). Prevalence of depression in patients with mild cognitive impairment: a systematic review and meta-analysis. JAMA Psychiat.

[74] Masters MC, Morris JC, Roe CM (2015). “Noncognitive” symptoms of early Alzheimer disease. Neurology.

[75] Slifer MA, Martin ER, Gilbert JR, Haines JL, Pericak-Vance MA (2009). Resolving the relationship between apolipoprotein E and depression. Neurosci Lett.

[76] Dissanayake AS, Tan YB, Bowie CR, Butters MA, Flint AJ, Gallagher D (2022). Sex modifies the associations of APOE ɛ4 with neuropsychiatric symptom burden in both at-risk and clinical cohorts of Alzheimer’s disease. J Alzheimers Dis.

[77] Kaufer DI, Cummings JL, Ketchel P, Smith V, MacMillan A, Shelley T (2000). Validation of the NPI-Q, a brief clinical form of the Neuropsychiatric Inventory. JNP.

