# Supplementary material for: Mild behavioral impairment in early Alzheimer’s disease and its association with APOE and BDNF risk genetic polymorphisms
Source: Alzheimers Res Ther. 2024 Jan 26;16:21. doi: 10.1186/s13195-024-01386-y (PMC10811933; doi:10.1186/s13195-024-01386-y)
Supplement: Supplementary file 1 — Additional file 1: Table S1. The association between MBI total score and APOE and BDNF polymorphism groups. Table S2.1. The association between MBI domain scores and APOE polymorphism groups. Table S2.2. The association between MBI domain scores and BDNF polymorphism groups. Table S2.3. The association between MBI domain scores and APOE and BDNF polymorphism groups. Table S3. The association between GDS-15 and APOE and BDNF polymorphism groups. Table S4. The association between BAI and APOE and BDNF polymorphism groups. [file 13195_2024_1386_MOESM1_ESM.docx]

**Supplementary material**

**Supplementary Table S1. The association between MBI total score and APOE and BDNF polymorphism groups**

|  | **Model 1.1** | | | **Model 1.2** | | | **Model 1.3** | | |
| --- | --- | --- | --- | --- | --- | --- | --- | --- | --- |
| *Predictors* | *Estimates* | *CI* | *p* | *Estimates* | *CI* | *p* | *Estimates* | *CI* | *p* |
| (Intercept) | 20.99 | -30.67 – 72.65 | 0.418 | 22.66 | -19.57 – 64.90 | 0.287 | 21.15 | -31.49 – 73.79 | 0.423 |
| age | 0.16 | -0.40 – 0.73 | 0.559 | 0.08 | -0.38 – 0.53 | 0.736 | 0.17 | -0.40 – 0.74 | 0.559 |
| sex | -2.17 | -7.30 – 2.95 | 0.398 | -2.22 | -6.94 – 2.50 | 0.349 | -2.56 | -7.97 – 2.85 | 0.345 |
| MMSE | -0.82 | -1.97 – 0.33 | 0.159 | -0.68 | -1.61 – 0.24 | 0.145 | -0.86 | -2.05 – 0.33 | 0.152 |
| BDNF | -1.29 | -6.92 – 4.34 | 0.646 |  |  |  | -1.68 | -12.31 – 8.96 | 0.753 |
| APOE |  |  |  | 0.52 | -4.78 – 5.82 | 0.846 | 1.75 | -6.11 – 9.61 | 0.656 |
| BDNF * APOE |  |  |  |  |  |  | 0.40 | -11.89 – 12.69 | 0.948 |
| Observations | 52 | | | 61 | | | 52 | | |
| R^2^ / R^2^ adjusted | 0.055 / -0.025 | | | 0.047 / -0.021 | | | 0.064 / -0.061 | | |

Notes: Estimates, unstandardized regression coefficients; MBI, mild behavioral impairment; CI, Confidence interval; MMSE, Mini-Mental State Examination; BDNF, Brain-Derived Neurotrophic Factor (2 groups); APOE, Apolipoprotein E (2 groups)

Model 1.1 tests the association between BDNF and MBI-C total score; Model 1.2 tests the association between APOE and MBI-C total score; and Model 1.3 tests the association between the interaction of BDNF * APOE and MBI-C total score.

**Supplementary Table S2.1. The association between MBI domain scores and APOE polymorphism groups**

|  | **Model 2.1.1** | | | **Model 2.1.2** | | | **Model 2.1.3** | | | | **Model 2.1.4** | | | | **Model 2.1.5** | | |
| --- | --- | --- | --- | --- | --- | --- | --- | --- | --- | --- | --- | --- | --- | --- | --- | --- | --- |
| *Predictors* | *Estimates* | *CI* | *p* | *Estimates* | *CI* | *p* | *Estimates* | *CI* | *p* | *Estimates* | | *CI* | *p* | *Estimates* | | *CI* | *p* |
| (Intercept) | 8.74 | -5.02 – 22.50 | 0.209 | 2.98 | -10.09 – 16.04 | 0.650 | 10.97 | -6.73 – 28.67 | 0.220 | -0.09 | | -3.47 – 3.29 | 0.960 | 0.07 | | -4.15 – 4.29 | 0.974 |
| age | -0.01 | -0.16 – 0.14 | 0.876 | 0.04 | -0.10 – 0.18 | 0.549 | 0.01 | -0.18 – 0.20 | 0.931 | 0.01 | | -0.03 – 0.05 | 0.558 | 0.03 | | -0.02 – 0.07 | 0.235 |
| sex | -1.15 | -2.69 – 0.39 | 0.140 | 0.25 | -1.21 – 1.71 | 0.735 | -1.11 | -3.09 – 0.87 | 0.265 | -0.02 | | -0.39 – 0.36 | 0.933 | -0.19 | | -0.67 – 0.28 | 0.411 |
| MMSE | -0.18 | -0.48 – 0.12 | 0.237 | -0.16 | -0.45 – 0.12 | 0.264 | -0.28 | -0.67 – 0.10 | 0.150 | -0.00 | | -0.08 – 0.07 | 0.899 | -0.05 | | -0.15 – 0.04 | 0.240 |
| APOE | 0.51 | -1.22 – 2.24 | 0.557 | 0.22 | -1.42 – 1.86 | 0.787 | 0.28 | -1.94 – 2.50 | 0.802 | -0.34 | | -0.76 – 0.08 | 0.114 | -0.15 | | -0.68 – 0.38 | 0.566 |
| Observations | 61 | | | 61 | | | 61 | | | | 61 | | | | 61 | | |
| R^2^ / R^2^ adjusted | 0.053 / -0.014 | | | 0.034 / -0.035 | | | 0.049 / -0.019 | | | | 0.060 / -0.007 | | | | 0.072 / 0.006 | | |

Notes: Estimates, unstandardized regression coefficients; MBI, mild behavioral impairment; CI, Confidence interval; MMSE, Mini-Mental State Examination; APOE, Apolipoprotein E (2 groups)

Model 2.1.1 tests the association between APOE and MBI-C motivation score; Model 2.1.2 tests the association between APOE and MBI-C affective dysregulation score; Model 2.1.3 tests the association between APOE and MBI -C impulse dyscontrol score; Model 2.1.4 tests the association APOE and MBI-C social inapropriateness score; Model 2.1.5 tests the association between APOE and MBI-C abnormal perception/thought score.

**Supplementary Table S2.2. The association between MBI domain scores and BDNF polymorphism groups**

|  | **Model 2.2.1** | | | **Model 2.2.2** | | | **Model 2.2.3** | | | | **Model 2.2.4** | | | | **Model 2.2.5** | | |
| --- | --- | --- | --- | --- | --- | --- | --- | --- | --- | --- | --- | --- | --- | --- | --- | --- | --- |
| *Predictors* | *Estimates* | *CI* | *p* | *Estimates* | *CI* | *p* | *Estimates* | *CI* | *p* | *Estimates* | | *CI* | *p* | *Estimates* | | *CI* | *p* |
| (Intercept) | 8.98 | -7.61 – 25.57 | 0.282 | 3.29 | -12.87 – 19.45 | 0.684 | 10.16 | -11.68 – 32.00 | 0.354 | -1.03 | | -5.06 – 3.00 | 0.610 | -0.41 | | -5.55 – 4.72 | 0.872 |
| age | -0.02 | -0.20 – 0.16 | 0.790 | 0.07 | -0.10 – 0.25 | 0.420 | 0.05 | -0.19 – 0.29 | 0.674 | 0.03 | | -0.01 – 0.08 | 0.145 | 0.04 | | -0.02 – 0.09 | 0.211 |
| sex | -1.01 | -2.65 – 0.64 | 0.225 | 0.18 | -1.42 – 1.78 | 0.822 | -1.07 | -3.23 – 1.10 | 0.327 | -0.07 | | -0.47 – 0.33 | 0.726 | -0.21 | | -0.72 – 0.30 | 0.406 |
| MMSE | -0.15 | -0.51 – 0.22 | 0.432 | -0.23 | -0.59 – 0.13 | 0.202 | -0.34 | -0.83 – 0.14 | 0.162 | -0.03 | | -0.12 – 0.06 | 0.456 | -0.06 | | -0.18 – 0.05 | 0.264 |
| BDNF | 0.17 | -1.64 – 1.97 | 0.855 | -0.69 | -2.45 – 1.07 | 0.434 | -0.75 | -3.13 – 1.62 | 0.526 | -0.14 | | -0.58 – 0.29 | 0.512 | 0.13 | | -0.43 – 0.69 | 0.636 |
| Observations | 52 | | | 52 | | | 52 | | | | 52 | | | | 52 | | |
| R^2^ / R^2^ adjusted | 0.045 / -0.037 | | | 0.053 / -0.027 | | | 0.055 / -0.025 | | | | 0.058 / -0.022 | | | | 0.086 / 0.008 | | |

Notes: Estimates, unstandardized regression coefficients; MBI, mild behavioral impairment; CI, Confidence interval; MMSE, Mini-Mental State Examination; BDNF, Brain-Derived Neurotrophic Factor (2 groups).

Model 2.2.1 tests the association between BDNF and MBI-C motivation score; Model 2.2.2 tests the association between BDNF and MBI-C affective dysregulation score; Model 2.2.3 tests the association between BDNF and MBI -C impulse dyscontrol score; Model 2.2.4 tests the association BDNF and MBI-C social inapropriateness score; Model 2.2.5 tests the association between BDNF and MBI-C abnormal perception/thought score.

|  | **Model 2.3.1~~MBI_mot~~** | | | **Model 2.3.2~~MBI_aff~~** | | | **Model 2.3.3~~MBI_imp~~** | | | **Model 2.3.4~~MBI_soc~~** | | | **Model 2.3.5~~MBI_hal~~** | | |
| --- | --- | --- | --- | --- | --- | --- | --- | --- | --- | --- | --- | --- | --- | --- | --- |
| *Predictors* | *Estimates* | *CI* | *p* | *Estimates* | *CI* | *p* | *Estimates* | *CI* | *p* | *Estimates* | *CI* | *p* | *Estimates* | *CI* | *p* |
| (Intercept) | 9.08 | -7.67 – 25.82 | 0.281 | 3.51 | -12.81 – 19.84 | 0.667 | 10.11 | -12.13 – 32.34 | 0.365 | -1.08 | -5.12 – 2.96 | 0.593 | -0.46 | -5.67 – 4.75 | 0.859 |
| age | -0.02 | -0.20 – 0.16 | 0.806 | 0.07 | -0.11 – 0.25 | 0.424 | 0.05 | -0.19 – 0.29 | 0.671 | 0.03 | -0.01 – 0.08 | 0.150 | 0.04 | -0.02 – 0.09 | 0.215 |
| sex | -1.23 | -2.95 – 0.50 | 0.159 | -0.02 | -1.70 – 1.66 | 0.979 | -1.14 | -3.42 – 1.15 | 0.321 | 0.01 | -0.41 – 0.42 | 0.971 | -0.18 | -0.72 – 0.35 | 0.494 |
| MMSE | -0.17 | -0.55 – 0.21 | 0.371 | -0.26 | -0.63 – 0.10 | 0.155 | -0.34 | -0.85 – 0.16 | 0.174 | -0.02 | -0.11 – 0.07 | 0.607 | -0.06 | -0.18 – 0.06 | 0.326 |
| BDNF | -0.03 | -3.41 – 3.36 | 0.987 | 0.56 | -2.73 – 3.86 | 0.732 | -1.70 | -6.19 – 2.80 | 0.451 | -0.27 | -1.09 – 0.54 | 0.501 | -0.24 | -1.29 – 0.81 | 0.647 |
| APOE | 1.00 | -1.50 – 3.50 | 0.426 | 1.26 | -1.18 – 3.70 | 0.303 | 0.11 | -3.21 – 3.43 | 0.946 | -0.40 | -1.00 – 0.20 | 0.189 | -0.22 | -1.00 – 0.55 | 0.563 |
| BDNF * APOE | 0.19 | -3.72 – 4.10 | 0.921 | -1.76 | -5.57 – 2.05 | 0.358 | 1.25 | -3.94 – 6.44 | 0.630 | 0.20 | -0.74 – 1.14 | 0.671 | 0.51 | -0.70 – 1.73 | 0.399 |
| Observations | 52 | | | 52 | | | 52 | | | 52 | | | 52 | | |
| R^2^ / R^2^ adjusted | 0.070 / -0.054 | | | 0.078 / -0.045 | | | 0.065 / -0.060 | | | 0.099 / -0.021 | | | 0.101 / -0.019 | | |

**Supplementary Table S2.3. The association between MBI domain scores and APOE and BDNF polymorphism groups**

Notes: Estimates, unstandardized regression coefficients; MBI, mild behavioral impairment; CI, Confidence interval; MMSE, Mini-Mental State Examination; BDNF, Brain-Derived Neurotrophic Factor (2 groups); APOE, Apolipoprotein E (2 groups)

Model 2.3.1 tests the association between the interaction of BDNF * APOE and MBI-C motivation score; Model 2.3.2 tests the association between the interaction of BDNF * APOE and MBI-C affective dysregulation score; Model 2.3.3 tests the association between the interaction of BDNF * APOE and MBI -C impulse dyscontrol score; Model 2.3.4 tests the association between the interaction of BDNF * APOE and MBI-C social inapropriateness score; Model 2.3.5 tests the association between the interaction of BDNF * APOE and MBI-C abnormal perception/thought score.

**Supplementary Table S3. The association between GDS-15 and APOE and BDNF polymorphism groups**

**Supplementary Table S3. The association between GDS-15 and APOE and BDNF polymorphism groups**

|  | **Model 3.1** | | | **Model 3.2** | | | **Model 3.3** | | |
| --- | --- | --- | --- | --- | --- | --- | --- | --- | --- |
| *Predictors* | *Estimates* | *CI* | *p* | *Estimates* | *CI* | *p* | *Estimates* | *CI* | *p* |
| (Intercept) | 0.79 | -10.72 – 12.30 | 0.891 | 1.21 | -8.79 – 11.21 | 0.809 | 0.77 | -10.92 – 12.46 | 0.895 |
| age | 0.02 | -0.10 – 0.15 | 0.727 | 0.03 | -0.08 – 0.14 | 0.597 | 0.02 | -0.11 – 0.15 | 0.742 |
| sex | 1.09 | -0.05 – 2.23 | 0.060 | 1.04 | -0.08 – 2.16 | 0.067 | 1.19 | -0.01 – 2.39 | 0.052 |
| MMSE | -0.07 | -0.32 – 0.19 | 0.593 | -0.06 | -0.28 – 0.16 | 0.594 | -0.06 | -0.32 – 0.20 | 0.653 |
| BDNF | 0.59 | -0.67 – 1.84 | 0.351 |  |  |  | 0.89 | -1.47 – 3.25 | 0.453 |
| APOE |  |  |  | -0.90 | -2.16 – 0.35 | 0.155 | -0.39 | -2.14 – 1.35 | 0.654 |
| BDNF * APOE |  |  |  |  |  |  | -0.37 | -3.10 – 2.35 | 0.783 |
| Observations | 52 | | | 61 | | | 52 | | |
| R^2^ / R^2^ adjusted | 0.119 / 0.044 | | | 0.091 / 0.026 | | | 0.133 / 0.017 | | |

Notes: Estimates, unstandardized regression coefficients; GDS-15, Geriatric Depression Scale, 15-item version; CI, Confidence interval; MMSE, Mini-Mental State Examination; BDNF, Brain-Derived Neurotrophic Factor (2 groups); APOE, Apolipoprotein E (2 groups)

Model 3.1 tests the association between BDNF and GDS-15 score; Model 3.2 tests the association between APOE and GDS-15 score; and Model 3.3 tests the association between the interaction of BDNF * APOE and GDS-15 score.

|  | **Model 4.1** | | | **Model 4.2** | | | **Model 4.3** | | |
| --- | --- | --- | --- | --- | --- | --- | --- | --- | --- |
| *Predictors* | *Estimates* | *CI* | *p* | *Estimates* | *CI* | *p* | *Estimates* | *CI* | *p* |
| (Intercept) | 2.98 | -39.43 – 45.39 | 0.888 | 17.11 | -17.27 – 51.50 | 0.323 | 3.46 | -37.18 – 44.10 | 0.865 |
| age | -0.13 | -0.59 – 0.34 | 0.587 | -0.15 | -0.52 – 0.22 | 0.434 | -0.13 | -0.58 – 0.31 | 0.542 |
| sex | 3.97 | -0.23 – 8.18 | 0.064 | 4.24 | 0.39 – 8.08 | 0.031 | 4.31 | 0.13 – 8.48 | 0.044 |
| MMSE | 0.27 | -0.68 – 1.21 | 0.570 | -0.09 | -0.84 – 0.66 | 0.814 | 0.25 | -0.66 – 1.17 | 0.579 |
| BDNF | 3.78 | -0.84 – 8.40 | 0.107 |  |  |  | 10.73 | 2.51 – 18.94 | **0.012** |
| APOE |  |  |  | -3.52 | -7.84 – 0.80 | 0.108 | 0.07 | -6.00 – 6.13 | 0.983 |
| BDNF * APOE |  |  |  |  |  |  | -9.29 | -18.78 – 0.20 | 0.055 |
| Observations  R^2^ / R^2^ adjusted | 52  0.117 / 0.042 | | | 61  0.113 / 0.049 | | | 52  0.226 / 0.123 | | |

**Supplementary Table S4. The association between BAI and APOE and BDNF polymorphism groups**

Notes: Estimates, unstandardized regression coefficients; BAI, Beck Anxiety Inventory; CI, Confidence interval; MMSE, Mini-Mental State Examination; BDNF, Brain-Derived Neurotrophic Factor (2 groups); APOE, Apolipoprotein E (2 groups)

Model 4.1 tests the association between BDNF and BAI score; Model 4.2 tests the association between APOE and BAI score; and Model 4.3 tests the association between the interaction of BDNF * APOE and BAI score.
